# Pituitary-derived small extracellular vesicles promote liver repair by its cargo miR-143-3p

**DOI:** 10.1038/s41598-024-67434-7

**Published:** 2024-07-18

**Authors:** Jia-Li Xiong, Yu-Xuan Wang, Jun-Yi Luo, Shu-Meng Wang, Jia-Jie Sun, Qian-Yun Xi, Ting Chen, Yong-Liang Zhang

**Affiliations:** 1 College of Animal Science, Guangdong Province Key Laboratory of Animal Nutritional Regulation, National Engineering Research Center for Breeding Swine Industry, State Key Laboratory of Livestock and Poultry Breeding, South China Agricultural University, Guangzhou, 510642 Guangdong China; 2 College of Medicine, Jiaxing University, Jiaxing, 314000 Zhejiang China

**Keywords:** Pituitary small extracellular vesicles, miR-143-3p, Liver, Proliferation, Repair, Biochemistry, Cell biology, Molecular biology

## Abstract

The small Extracellular vesicles (sEV) has been recognized to be significant for intercellular communication due to their ability to transfer important cellular cargoes like miRNAs through circulation. The pituitary gland has not been clearly known about the role of its secreted sEV under normal physiological conditions. And Liver disease is a global public health burden. The present study is the first to investigate the effect of pituitary sEV on the liver. Sequencing and qRT-PCR revealed miR-143-3p is one of the richest in the pituitary sEV. MiR-143 Knockout (KO) mice resulted in a remarkable decrease in insulin-like growth factor 1 (IGF-1) levels and a significant increase in insulin-like growth factor binding protein 5 (IGFBP5) levels along with a reduction in liver primary cell growth. More importantly, compared with miR-143-KO-sEV, WT-sEV possesses a more robust capacity to improve miR-143 KO mice liver repair through the Wnt/β-catenin pathway after an acute injury caused by carbon tetrachloride (CCl_4_). Our results indicate that pituitary-derived sEV promotes hepatocyte proliferation and liver repair by its cargo miR-143-3p and provides new insight into the regulation mechanism of the pituitary-liver axis, and open a new window for endocrine regulation by using sEV.

## Introduction

The transmission and response of various signals between cells and tissues is fundamental in maintaining whole-body homeostasis. Small Extracellular Vesicle (sEV) has been documented as a major vehicle of intercellular communication in recent years. It is a type of nano-scale vesicles released by cells into the extracellular environment upon endocytic fusion with the plasma membrane and plays an essential role in intercellular communication in normal and diseased states^1–6^. It carries different types of cargos, such as proteins, lipids, messenger RNA (mRNA), non-coding RNA (ncRNA), such as microRNA (miRNA)^7^. Multiple studies suggest that sEV can exert its regulation by transporting miRNA to the recipient cells^8–12^. Hence, extracellular miRNAs are emerging as a new messenger and effector in intercellular communication. More importantly, sEV was indicated to possibly serve important roles in endocrine regulation. sEV from thymic epithelial cells was able to participate in the thymocyte selection process^13^. The beta-cell sEV has been reported to be involved in the cross-talk with endothelial cells or lymphocytes^14,15^. Moreover, sEV is also an important mediator of the thyroid in immune modulation^16–18^.

The pituitary gland, regarded as the master gland, plays pivotal roles in the modulation of key physiological functions. Up to now, reports of pituitary sEV have always been limited to pituitary adenoma^19–22^. Studies have shown that sEV-derived lncRNA H19, miR-149-5p and miR-99a-3p inhibit the progress of pituitary adenoma and hsa-miR-21-5p in pituitary adenoma promotes abnormal bone formation in acromegaly. More interestingly, a study demonstrated that adenoma pituitary sEV may serve as nonhormonal pituitary-derived messengers mediating intercellular communication^21^. Unfortunately, there is very rare information on sEV from normal pituitary. Our previous study has explored the ncRNAs in anterior pituitary sEV from the Duroc swine model^23^, and the bioinformatics analyses revealed that pituitary sEV can be broadly involved in the signaling process and biological regulation^23^. If pituitary sEV is a new way to deliver signals to other cells, it remains an academically significant question in pituitary physiology. Thus, we hypothesized that the pituitary gland regulates its target cells by not only secreting hormones but also sEV as well.

As a major site for both nutrition and xenobiotic metabolism, the liver plays an important role in metabolic homeostasis. Nevertheless, previous research on the regulation of the pituitary to the liver was mostly through hormones, and little is known about the pituitary sEV regulatory pathway. Studies have demonstrated that some components, like miRNA inside sEV, possess the capacity to enhance liver proliferation and regulate endogenous repair^24–29^. Therefore, in this study, we aimed to explore the role of pituitary sEV in hepatocyte proliferation and the key miRNAs involved in this process.

Herein, for the first time, we investigated the regulatory role of pituitary sEV as a mode of intercellular communication with hepatocytes. We demonstrate that sEV produced by the pituitary gland promotes hepatocyte proliferation in vitro and the liver repair of Carbon tetrachloride (CCl_4_)-induced acute liver injury in vivo. The mechanism of this effect is due to the miR-143-3p in sEV released from pituitary cells, by promoting liver repair through the Wnt/β-catenin pathway. Our study reveals a novel mechanism by which the pituitary can remotely regulate the liver via pituitary sEV-derived miRNA.

## Materials and methods

### Animal model

The miR-143 knockout mice (miR-143 KO, FVB) were generated by Cyagen Biosciences (Guangzhou, China) Inc. using the CRISPR/Cas9 system. Mice used for experiments were 6–8 weeks of age and housed under standard conditions (12:12 light/dark cycle).

### Cell and tissue block culture

The normal rat hepatocytes BRL 3A (category number GNR10) was purchased from The Cell Bank of Culture Collection of Chinese Academy of Sciences (Shanghai Institute of Cell Biology). BRL 3A cell was cultured in DMEM basic (Gibco, USA) medium supplemented with 10% fetal bovine serum (FBS) (Gibco, USA) and 1% penicillin/streptomycin (Gibco, USA). The rat pituitary MMQ cell line (ATCC, CRL-10609) was cultured in DMEM/F12 (Gibco, USA) medium supplemented with 2.5% FBS, 15% horse serum (Hyclone, USA) and 1% penicillin/streptomycin (Gibco, USA). 48 h before sEV harvest, the cell culture medium was replaced with a fresh EV-depleted serum medium. EV-depleted serum was prepared by subjecting serum to ultracentrifugation (Optima XE, Beckman Coulter) at 120,000 × g for 18 h at 4 °C according to the literature^30^.The mouse pituitary glands were removed under sterile conditions after being sacrificed by cervical dislocation, rinsed in phosphate-buffered saline (PBS), cut up into 1 mm^3^ pieces, and cultured in serum-free DMEM/F12 (Gibco, US) for 48 h.

### Isolation and characterization of sEV

Ultracentrifugation (Beckman 32Ti rotor) was used to isolate sEV from the collected conditioned medium followed the guideline of MISEV2018 ^31–33^. The obtained pellet was resuspended in PBS and stored at − 80 °C. We assessed sEVs protein concentration using the BCA Protein Assay Kit (Thermo Fisher Scientific, Waltham, MA, USA). Morphology of sEV were examined with transmission electron microscope. A drop of sEV suspension (about 10 μL) was fixed on a formvar coated copper grid for 2 min, washed briefly in ultrapure water, negatively stained with 1% uranyl acetate, and observed by transmission electron microscopy (TEM; JEM-2000EX; Jeol, Tokyo, Japan) at an acceleration voltage of 80 kV. NTA measurements were performed using a NanoSight LM10 instrument (NanoSight, Amesbury, UK). The samples were diluted with PBS and injected into the LM unit (approximately 300 μl) with a 1 ml sterile syringe. The capturing settings (shutter and gain) and analyzing settings were manually set according to the protocol. The NanoSight LM10 recorded 60 s sample videos which were than analyzed with the Nanoparticle Tracking Analysis (NTA) 2.0 Analytical software release version build 0125. The sEVs markers (CD9, CD63 and TSG101 were used as positive control whereas the he endoplasmic reticulum protein Calnexin was used as negative control in Western blot analysis.

### Total RNA extraction, RNA-Seq library preparation, sequencing and data analysis

We extracted total RNA from EV suspension samples using Trizol reagent (Invitrogen, Carlsbad, CA) according to the manufacturer’s instruction. RNA quantity and quality were assessed using the RNA 6000 Nano Assay Kit of the Agilent Bioanalyzer 2100 system (Agilent Technologies, Inc., Santa Clara, CA). A total amount of 3 μg total RNA per sample was used as input material for the small RNA library. Sequencing libraries were generated using NEBNext® Multiplex Small RNA Library Prep Set for Illumina® (NEB, United States). After cluster generation, the library preparations were sequenced on an Illumina Hiseq 2500/2000 platform and 50 bp single-end reads were generated at the Novogene Bioinformatics Institute (Beijing, China). Raw data (raw reads) of fastq format were firstly processed through custom perl and python scripts at first. Clean reads were obtained by removing reads with poly-N,5′ adapter contaminants, poly A or T or G or C, those without 3’ adapter or the insert tag, and low-quality reads from raw data. Q20, Q30, and GC content of the raw datas were calculated at the same time. High-quality data were used for subsequent analyses. The small RNA tags were mapped to reference sequence by Bowtie^34^ without mismatch to analyze their expression and distribution on the reference. Mapped small RNA tags were used for searching known miRNA. Mirbase20.0 was used as reference, and modified software mirdeep2^35^ and srna-tools-cli were used to obtain the potential miRNA and draw the secondary structures. The characteristics of the hairpin structure of miRNA precursor can be used to predict novel miRNA. The available software miREvo^36^ and mirdeep2^35^ were integrated to predict novel miRNA through exploring the secondary structure, the Dicer cleavage site, and the minimum free energy of the small RNA tags unannotated in the former steps.

### Cell viability assay

Cell proliferation was evaluated using Cell counting kit-8 (CCK8; EZBioscience, EZB-CK8) method, 5-ethynyl-2ʹ-deoxy uridine (EdU; Beyotime Biotechnology, C0071S) incorporation assay. First, the rate of cell proliferation was determined with CCK-8 kit according to the manufacturer’s instructions. The number of viable cells was assessed by measuring the absorbance at 450 nm using a Synergy 2 Multi-Mode Reader (Bio Tek Instruments, Inc., Winooski, VT, USA). Secondly, DNA synthesis was examined with EdU incorporation assay to evaluate cell proliferation. The EdU positive cells were counted and normalized by the total number of Hoechst 33,342 stained cells.

### Transfection of miR-143-3p mimic and inhibitor

BRL 3A cells in 12-well plates (about 4 × 10^5^cell/well) were cultured and transfected with miR-143-3p mimics, or miR-143-3p inhibitor, or their corresponding negative controls with Lipofectamine 2000 (Invitrogen, Carlsbad, CA, USA) following the manufacturer’s introduction. After 6 h, the culture medium was replaced to remove the transfection reagent and the cells were incubated in complete culture medium for 48 h before collection.

### RNA extraction and quantitative polymerase chain reaction (qPCR)

Total RNA was extracted from cells and tissues using Trizol reagent (Invitrogen, Carlsbad, CA, USA) according to the manufacturer’s protocol. The cDNAs were obtained by Color Reverse Transcription Kit (with gDNA remover) (EZBioscience, Roseville, CA, USA). All qPCR experiments were performed on a real-time PCR machine (Bio-Rad Laboratories, Inc., Hercules, CA, USA) and specific primers of the genes of interest, and amplification efficiencies were checked by standard curves. Gene expression was quantified by the comparative cycle threshold (Ct) method. The target gene expression was normalized to those of β-actin, while the relative miRNA levels were normalized to the U6 or cel-miR-39 in sEV and calculated as 2^−(ΔΔCT)^.

### Western blot

Total protein of indicated tissues, cells or sEV was extracted using RIPA lysis buffer (Beyotime Institute of Biotechnology, Shanghai, China) containing 1 mM phenyl methane sulfonyl fluoride (PMSF). The protein concentrations were determined using the BCA Protein Assay Kit (Thermo Fisher Scientific, Waltham, MA, USA) according to the manufacturer’s instructions. Equal amounts of total protein were separated by SDS-PAGE and transferred to a PVDF membrane in a tris–glycine methanol buffer. Primary antibodies used include anti-CD9 (Sangon Biotech), anti-CD63 (Sangon Biotech), TSG101 (Zen Biotech), anti-PCNA (Zen Biotech), anti-IGFBP5 (Zen Biotech), anti-IGF-1 (Zen Biotech), anti-β-catenin (Bioss), anti-TCF-4 (Zen Biotech) and anti-Tubulin (Bioworld). Membranes were washed and incubated with either anti-mouse or anti-rabbit antibody conjugated to horseradish peroxidase (HRP) for 30 min to 1 h at room temperature. The membranes were incubated with ImmobilonTM Western Chemiluminescent HPR Substrate (Millipore, Burlington, WA, USA) and scanned with a FlourChem M Fluorescent Western Imaging System (Protein Simple, Santa Clara, CA, USA).

### ELISA

The concentrations of IGF-1 IGFBP5 were measured with ELISA kits purchased from Nanjing Jiancheng Bioengineering Institute. ELISA was performed according to the manufacturer’s instructions. Color alterations in the wells were read using the 96-well microplate reader (BioTek Instruments).

#### Pituitary sEV-labeling

Pituitary sEV were labeled with PKH67 Green Fluorescent Cell Linker Kit as previously reported^33,37^. 100 ul pituitary sEV suspension and 4 ul PKH67 were respectively added to 1 ml diluent C solution, gently mixed and incubated at room temperature for 5 min. 2 ml 0.5% BSA/PBS was added to bind excess dye. The labeled sEV was re-enriched at 120000 g for 1.5 h and re-suspended in 100ul PBS and used for uptake experiments.

#### Isolation and characterization of primary mouse hepatocytes

Primary mouse hepatocytes were isolated from WT and miR-143 KO mice with two-step collagenase perfusion method as described previously^38–41^. and freshly-isolated primary mouse hepatocytes were cultured in DMEM supplemented with 10% FBS. Primary mouse hepatocytes were characterized by the morphological characteristics and the relatively specific marker CK18^42^.

#### Acute liver injury with CCl_4_ injection and detection of serological markers

miR-143 KO mice were randomly divided into three group with each group containing three mice. Mice were intraperitoneally injected with a 0.3% solution of CCl_4_ in sterile olive oil (Sangon Biotech) at dose of 10 ul/g animal weight^43,44^. After that, mice were immediately injected intravenously PBS (vehicle control), miR-143 KO-sEV (100 ug) or WT-sEV (100 ug) from mice pituitary for 24 h. Liver samples were harvested from several lobes and either fixed in 4% paraformaldehyde or snap frozen in liquid nitrogen and stored at − 80 °C until use. Serum was collected by centrifugation of eyeball blood for testing Aspartate Transaminase (AST) and Alanine transaminase (ALT) (Nanjing Jiancheng Bioengineering Institute) according to the manufacturer’s protocols.

#### Statistical analysis

Data are expressed as mean ± SEM. Student’s t-test was used for two group comparison, and one-way ANOVA was used among more than two groups. Differences were considered significant when *P* < 0.05.

### Ethics approval and informed consent to participate

The animal study was reviewed and approved by the Institutional Animal Care and Use Committee of South China Agricultural University, China. All animal experimentation complied with the laboratory animal management and welfare regulations approved by the Standing Committee of Guangdong People’s Congress (Guangzhou), China. Ethical code number: SCAU-AEC-2010-0416.

## Results

### Characterization of MMQ-sEV and effects on BRL 3A cells proliferation

**Figure 1:**
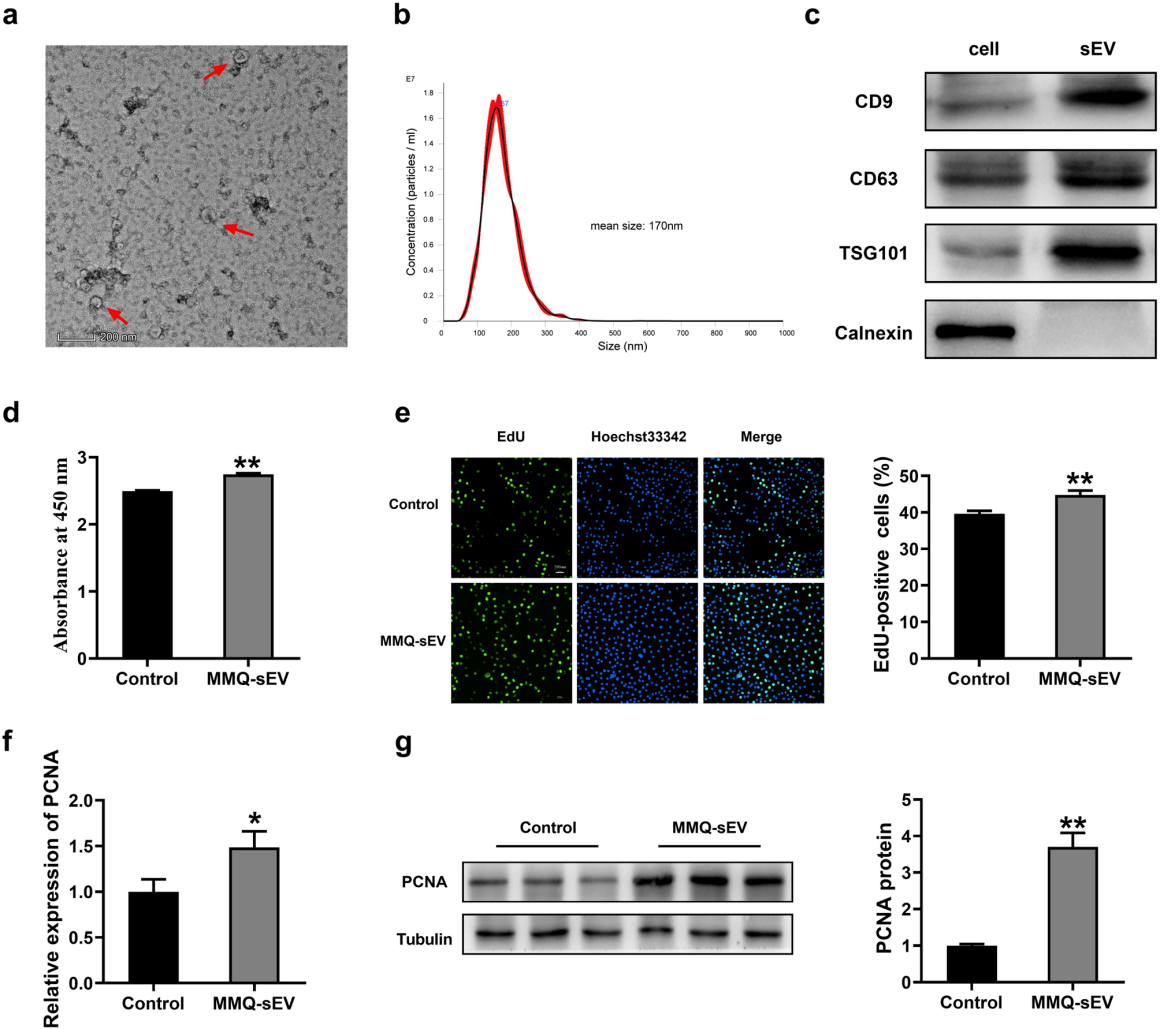
Characterization of MMQ-sEV and its effects on BRL 3A cells proliferation. (**a**) Analysis of MMQ-sEV by transmission electron microscopy. (**b**) Nanoparticle tracking analysis of size distribution of isolated MMQ-sEV. (**c**) Western Blot analysis showing the biomarkers of sEV including CD9, CD63 and TSG101. Calnexin was used as negative control. (**d**–**g**) Effects of MMQ-sEV on BRL-3A cells proliferation. The BRL 3A cells proliferation was evaluated with (**d**) CCK8 kits, (**e**) EdU-positive cells, (**f**) PCNA mRNA and (**g**) PCNA protein expression.

MMQ-sEV was harvested by ultracentrifugation conducted as described in the methods guided by MISEV2018^31–33^. Transmission electron microscopic (TEM) images revealed that MMQ-sEV had cup-like morphology (Fig. 1a). The distribution curve of the particle size of MMQ-sEV was determined by nanoparticle tracking analysis (NTA) (Fig. 1b), and the mean nanoparticle size of isolated MMQ-sEV is 170 ± 0.7 nm. In addition, sEV-associated protein makers, two transmembrane proteins (CD9 and CD63) and a cytosolic protein (TSG101) were found in MMQ-sEV, while a negative protein marker, an endoplasmic reticulum-residing protein (Calnexin) was not detected (Fig. 1c). The above results indicate the successful isolation of sEV from MMQ cells.

To explore whether MMQ-sEV had any modulatory effects on cell proliferation, MMQ-sEV (5 ug/ml/d, about 1 × 10^9^particles) was added to the culture medium of BRL 3A. Seventy-two hours later, the cell proliferation was evaluated with CCK8 kits and EdU staining, further confirmed by the detection of PCNA mRNA and protein expression. Interestingly, MMQ-sEV significantly promoted the proliferation of BRL-3A cells (Fig. 1d–g). This sparked our interest in exploring the mechanism of MMQ-sEV on hepatocyte proliferation.

### MiR-143 levels and effects of miR-143-3p on BRL-3A cells proliferation

**Figure 2:**
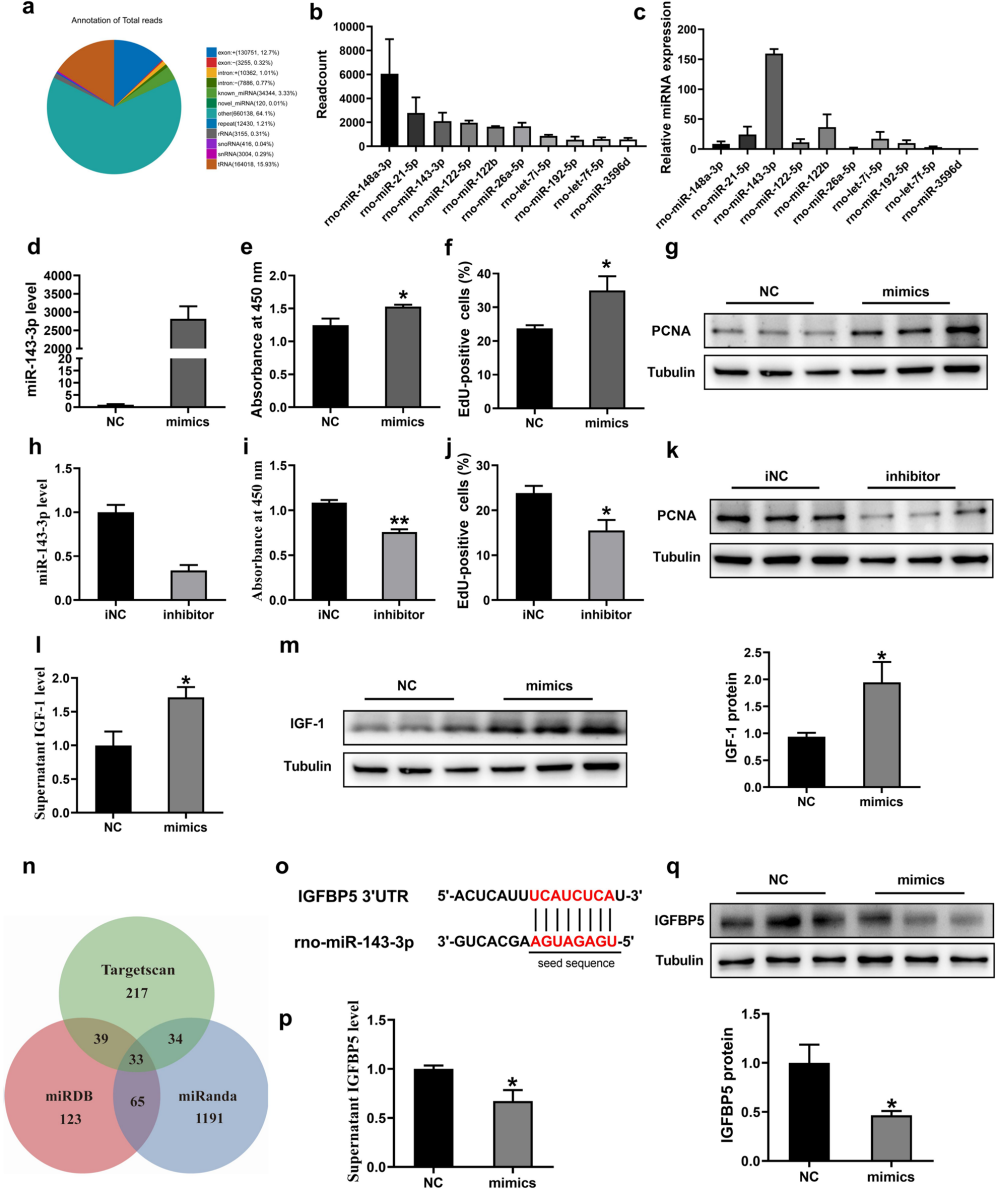
MiR-143 levels and effects of miR-143-3p on BRL-3A cells proliferation. (**a**) Annotation of total reads. (**b**) The top 10 miRNAs in RNA-seq. (**c**) miR-143-3p highly expressed in MMQ-sEV. (**d**) Relative expression of miR-143-3p following transfection with NC and miR-143-3p mimics(n = 6) (**e**–**g**) Effects of miR-143-3p mimics or its negative control on BRL 3A cells proliferation. The measurements were carried out 48 h after treatment. (e) CCK8 detection. (**f**) Quantification of EdU (percentage of EdU + /Hoechst +). (**g**) PCNA protein expression (n = 3). (**h**) Relative expression of miR-143-3p following transfection with iNC and miR-143-3p inhibitor (n = 6) (**i**–**k**) Effects of miR-143-3p inhibitor or its negative control on BRL 3A cells proliferation. (**i**) CCK8 kits. (**j**) Quantification of EdU (percentage of EdU + /Hoechst +). (**k**) PCNA protein expression (n = 3). (**l**) IGF-1 protein levels in supernatants of BRL 3A cells after transfected miR-143-3p mimics (n = 5). (**m**) Effects of miR-143-3p mimics on IGF-1 protein expression (n = 6) (**n**) Bioinformatic analysis using miRDB, miRanda and Targetscan to predict the possible target gene of miR-143-3p. (**o**) Sequence of rno-miR-143-3p binding site in the 3’UTR of IGFBP5. (p) IGFBP5 protein levels in supernatants of BRL 3A cells after transfected miR-143-3p mimics (n = 5). (**q**) Effects of miR-143-3p mimics on IGFBP5 protein expression in BRL 3A cells (n = 3).

RNA-sequencing technology was used to characterize the miRNAs from MMQ-sEV. A total of 420 miRNAs were obtained, 380 of which are known miRNAs and 40 were novel miRNAs. We analyzed the annotation of total reads (Fig. 2a) and the top10 miRNAs (Fig. 2b) were selected and validated by qRT-PCR. Interestingly, miR-143-3p was the third most highly expressed in sequencing but the top one miRNA in qPCR detection (Fig. 2c). Accordingly, miR-143-3p was found to be highly expressed in EVs of swine anterior pituitary in our preliminary study^23^. Based on the above results, we calculated that miR-143-3p might play an important role in MMQ-sEV-mediated regulation.

The results of MMQ-sEV RNA-sequencing and bioinformatics analysis also drew our interest in the potential role of miR-143-3p in promoting BRL 3A proliferation. To validate this conjecture, we further investigated the effect of miR-143-3p on BRL 3A cell proliferation. The efficiency of miR-143-3p transfection was confirmed by the dramatically upregulated miR-143-3p in BRL 3A (Fig. 2d), and this upregulation significantly facilitated BRL 3A cells proliferation (Fig. 2e–g), while miR-143-3p inhibitor reduced the expression of miR-143-3p and attenuated cells proliferation (Fig. 2h–k). These results indicate that miR-143-3p promotes BRL 3A proliferation and supports our findings in the sEV.

At the same time, the levels of IGF-1 in supernatant and cells were both significantly increased after overexpression of miR-143-3p (Fig. 2l–m). Previous studies have confirmed that IGF-1 promotes hepatocyte proliferation, differentiation, cell cycle progression and prolonged survival^45–47^. Moreover, the online bioinformatics tools miRDB (http://mirdb.org/), miRanda (www.microrna.org) and TargetScan (http://targetscan.org/) were used to predict the target genes of miR-143-3p and there were 33 identical genes in three groups (Fig. 2n). IGFBP5, a negative regulator of IGF-1 signaling, binds and sequesters IGF ligands^48^. Furthermore, there was a miR-143-3p binding site in the 3′UTR of IGFBP5 (Fig. 2o) and it has been validated in numerous studies^49–51^, and miR-143-3p can regulate IGF-1 signaling through IGFBP5 to regulate cell proliferation^49^. Then, we examined the expression of IGFBP5 after transfection of miR-143-3p mimics. The results showed that the expression of IGFBP5 was profoundly decreased, whether in cells or supernatant after miR-143-3p overexpression (Fig. 2p,q). The above results confirm that miR-143-3p can increase IGF-1 by reducing IGFBP5 to promote BRL 3A proliferation.

### miR-143-3p inhibitor reverse promotion of MMQ-sEV in BRL 3A proliferation

**Figure 3:**
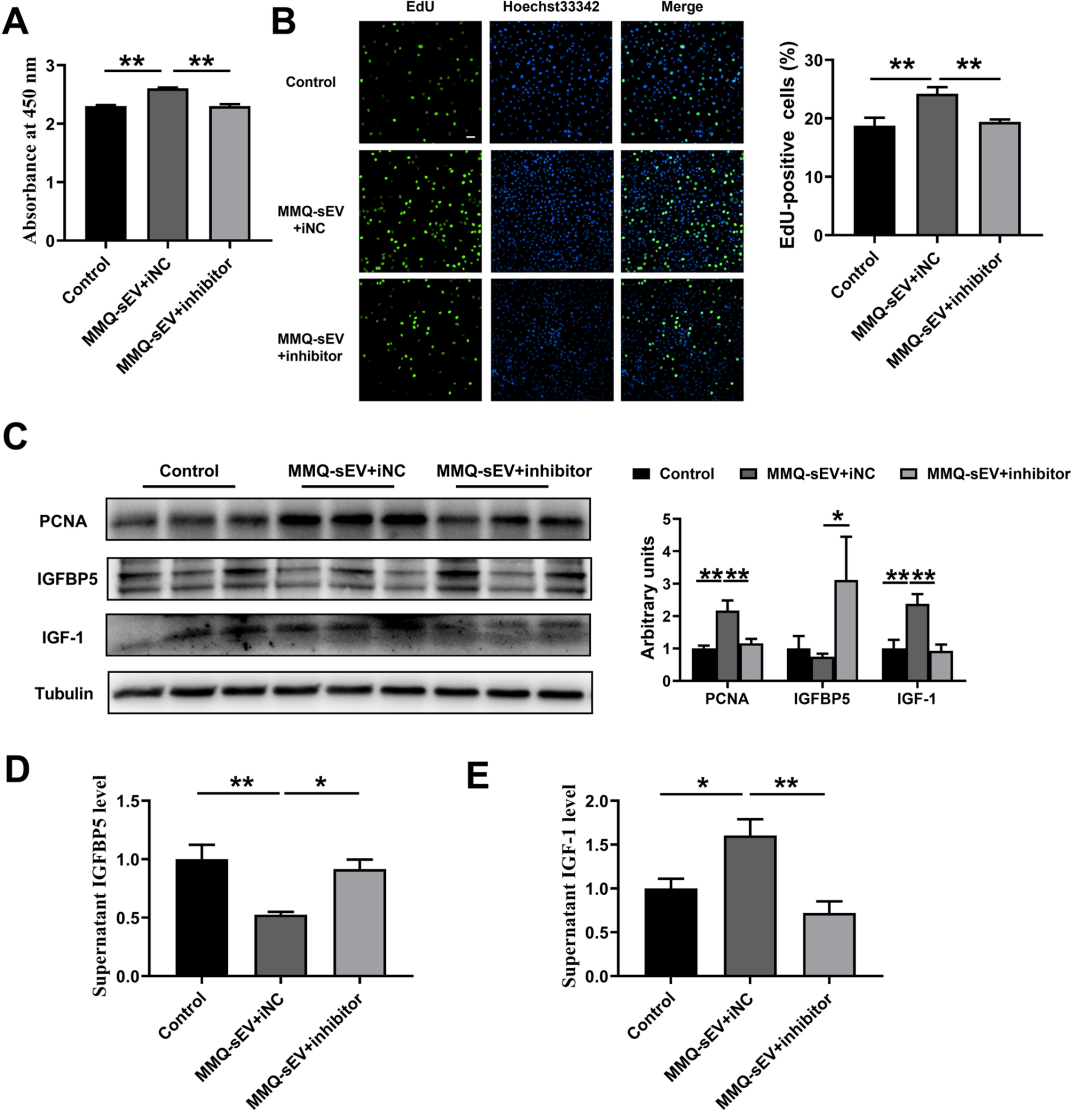
miR-143-3p inhibitor reverse promotion of MMQ-sEV on BRL 3A proliferation. (**a**) CCK8 kits. (**b**) Quantification of EdU (percentage of EdU + /Hoechst +). (**c**) Western blot analysis of PCNA, IGFBP5 and IGF-1 (n = 6). (**d**) IGFBP5 protein levels in supernatants of BRL-3A cells (n = 4). (**e**) IGF-1 protein levels in supernatants of BRL-3A cells (n = 4).

In "Characterization of MMQ-sEV and effects on BRL 3A cells proliferation" section, the promotion of BRL 3A proliferation by MMQ-sEV was found to be significant. Subsequently, the impact of the miR-143-3p inhibitor on this promotion was investigated. BRL 3A cells were subjected to treatment with Control (PBS + iNC), MMQ-sEV + iNC or MMQ-sEV + miR-143-3p inhibitor. It was observed that the inclusion of the miR-143-3p inhibitor reversed the facilitation of BRL 3A proliferation induced by MMQ-sEV (Fig. 3a–c). Additionally, MMQ-sEV notably enhanced the expression of IGF-1 by suppressing the protein expression of IGFBP5, and the miR-143-3p inhibitor could reverse this trend (Fig. 3c–e). These finding presented offer additional support for the notion that MMQ-sEV may enhance the proliferation of BRL 3A, potentially through the inclusion of miR-143-3p.

### MiR-143 knockout inhibits hepatocyte proliferation

**Figure 4:**
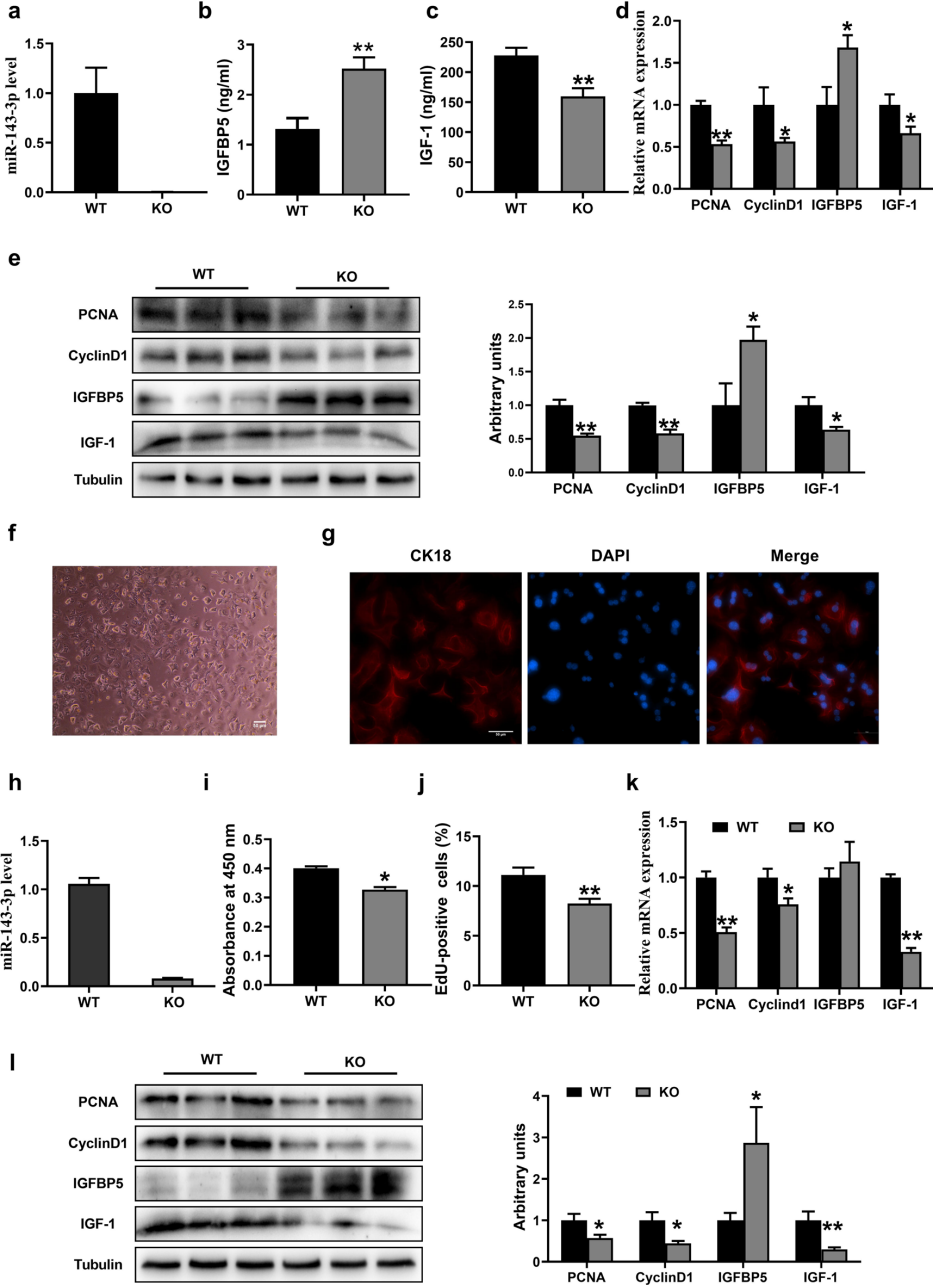
MiR-143 knockout inhibits hepatocyte proliferation (**a**) miR-143-3p levels in WT and miR-143 KO mice liver (n = 6). (**b**) Serum IGFBP5 level of WT and miR-143 KO mice (n = 6). (**c**) Serum IGF-1 level of WT and miR-143 KO mice (n = 6). (**d**) mRNA expression quantified by qRT-PCR (n = 6). (**e**) Western blot analysis of PCNA, CyclinD1, IGFBP5, IGF-1 in WT and KO mice liver (n = 6). (**f**) Cell morphology was detected by a microscope. (**g**) CK18 immunofluorescence staining. (**h**) miR-143-3p levels in WT and miR-143 KO primary hepatocytes (n = 8). (**i**) The proliferation was analyzed with CCK8 assay and (**j**) EdU quantification. (k) PCNA, CyclinD1, IGFBP5, IGF-1 mRNA expression quantified by qRT-PCR in WT and miR-143-3p KO hepatocytes (n = 8). (**l**) Western blot analysis of PCNA, CyclinD1, IGFBP5, IGF-1 in WT and miR-143 KO primary hepatocytes (n = 6).

To investigate the impact of miR-143-3p on IGF-1 signaling and hepatocyte proliferation, a mouse model with a knockout of miR-143 was successfully created using CRISPR/CAS9 technology, as previously outlined^52^. The miR-143-3p expression in the liver was quantified by using qRT-PCR and found to be dramatically down-regulated in KO mice (Fig. 4a). Interestingly, miR-143 KO mice showed a significantly higher level of IGFBP5 measured both in serum and the liver compared with wild-type (WT) mice (Fig. 4b,d–e). Oppositely, IGF-1 expression was decreased significantly (Fig. 4c–e). In addition, miR-143 KO reduced the expression of proliferation marker PCNA and cell cycle-associated protein Cyclin D1 in the liver (Fig. 4d,e).

We further verified the effect of miR-143 KO on hepatocyte proliferation in vitro. Hepatocytes were isolated by collagenase perfusion of the liver from WT and miR-143 KO mice, as described previously^38,39^. The isolated cells were identified by cell morphology (Fig. 4f) and means of immunofluorescence staining with antibodies against the specific marker CK18^42^ (Fig. 4g). Then, we analyzed the expression of miR-143-3p and cell proliferation of primary cells from two types of mice. As expected, the miR-143 KO hepatocytes grew more slowly (Fig. 4h–j) and showed higher levels of IGFBP5 and lower levels of IGF-1 (Fig. 4k–l) than WT cells. Taken together, these results strongly demonstrate that miR-143-3p plays a vital role in promoting hepatocyte proliferation.

### Wild type pituitary sEV promotes proliferation of miR-143 KO mouse hepatocytes

**Figure 5:**
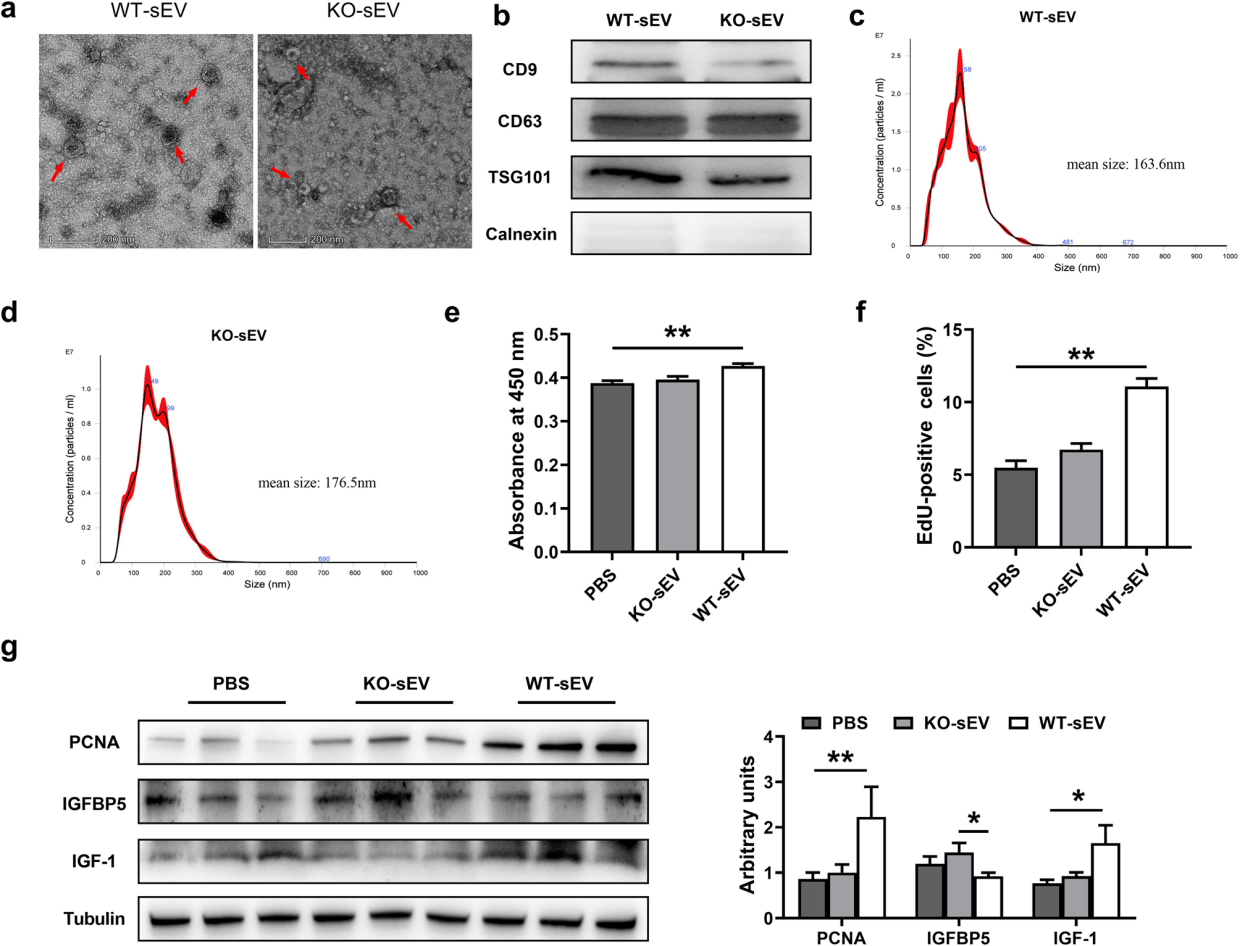
Wild type pituitary sEV promotes proliferation of miR-143 KO mouse hepatocytes (**a**) TEM images of pituitary sEV from WT and miR-143-KO mice. (**b**) Western blot analysis of CD9, CD63, TSG101 and Calnexin in WT-sEV and miR-143 KO-sEV. (**c**) Size distribution of WT-sEV. (**d**) Size distribution of miR-143 KO-sEV. The hepatocytes proliferation was evaluated with (**e**) CCK8 kits, (**f**) EdU-positive cells. (**g**) Expression of PCNA, IGFBP5 and IGF-1 protein with different treatment in miR-143KO hepatocytes (n = 6).

We prepared pituitary sEV from both wild-type and miR-143KO mice by the method described above. As visualized by TEM, both sEV appeared as round and cup-shaped vesicles (Fig. 5a). Western blot assay and NTA confirmed that miR-143 KO-sEV shares the expression of marker proteins (CD9, CD63 and TSG101) and diameter with WT-sEV (Fig. 5b–d).

We further carried out in vitro test with primary miR-143 KO hepatocytes. The results clearly showed that WT-sEV had a higher capability to promote cell proliferation compared with miR-143 KO-sEV in primary liver parenchymal (Fig. 5e–g). At the same time, the expression of IGF-1 was significantly up-regulated in comparison with the control group, and IGFBP5 was correspondingly in an opposite trend (Fig. 5g). The above results establish that pituitary sEV promotes hepatocyte proliferation by its cargo miR-143-3p.

### Wild-type pituitary sEV relieves liver injury in the miR-143 knockout mouse model by its cargo miR-143-3p

**Figure 6:**
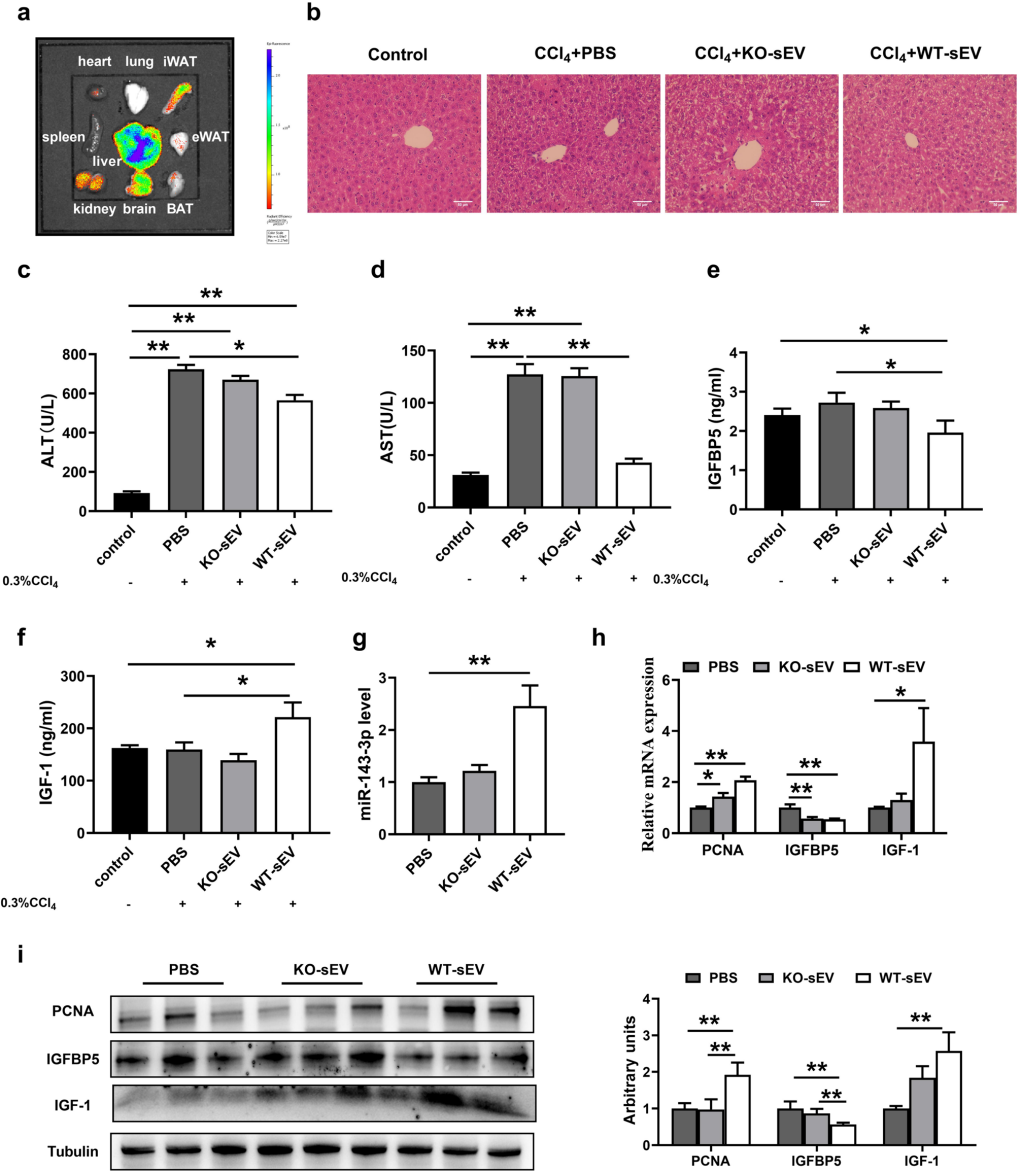
Wild-type pituitary sEV relieves liver injury in the miR-143 knockout mouse model by its cargo miR-143-3p. (**a**) Ex vivo imaging of major organs dissected after i.v. injection (**b**) Representative H&E staining images of the miR-143 KO mice livers from different groups. (**c**) Serum ALT levels (**d**) Serum AST levels. (**e**) Serum IGFBP5 levels. (**f**) Serum IGF-1 levels. (**g**) Relative expression of miR-143-3p after different treatment. (**h**) mRNA expression quantified by qRT-PCR (n = 6). (**i**)Western blot analysis of PCNA, IGFBP5, IGF-1 proteins in the livers of 143 KO mice from each group (n = 6).

To confirm the role of miR-143-3p from pituitary sEV on the liver, an in vivo test was conducted. In addition, to explore the distribution of pituitary sEV in vivo after injection via the tail vein, the biodistribution of pituitary sEV was imaged by labeling sEV with PKH-67. Ex vivo imaging of the dissected organs showed that fluorescence was mainly distributed in the liver (Fig. 6a). Furthermore, fluorescence can also be detected in the kidney and iWAT.

Then, a mouse liver injury model induced by 0.3%CCl_4_ was employed to explore the function of pituitary sEV, and its cargo miR-143-3p. In the following experiments, immediately after intraperitoneal injection 0.3% CCl_4_ into miR-143 KO mice, miR-143 KO-sEV or WT-sEV or PBS was injected into the tail vein. Twenty-four hours after injection, mice were sacrificed for sampling. The histopathological analysis showed that the liver of the PBS group was severely damaged, with an exhibition of dilation of the sinusoidal space with inflammatory cell infiltration and necrosis (Fig. 6b). Notably, the WT-sEV-treated group showed less cell necrosis, as well as more complete tissue structure than the miR-143 KO-sEV and PBS group did (Fig. 6b). Compared with the PBS-treated group, WT-sEV treatment, but not miR-143 KO-sEV treatment, significantly reduced the serum levels of alanine transaminase (ALT) and aspartate transaminase (AST), two important liver indicators (Fig. 6c,d). The above results indicate a better recovery of liver injury of WT-sEV than that of miR-143 KO-sEV.

In addition, the serum IGFBP5 level was obviously decreased by WT-sEV treatment (Fig. 6e), while IGF-1 was increased (Fig. 6f) following the elevated level of miR-143 (Fig. 6g). Moreover, the changes in the expression of IGFBP5 and IGF-1 in the liver were fully consistent with that in serum, and the expression level of PCNA was also significantly increased in the WT-sEV treatment group (Fig. 6h,i). The results above profoundly prove the idea that pituitary sEV relieves liver injury by its cargo miR-143-3p.

### MiR-143-3p in pituitary sEV promote liver repair via the Wnt/β-catenin pathway

**Figure 7:**
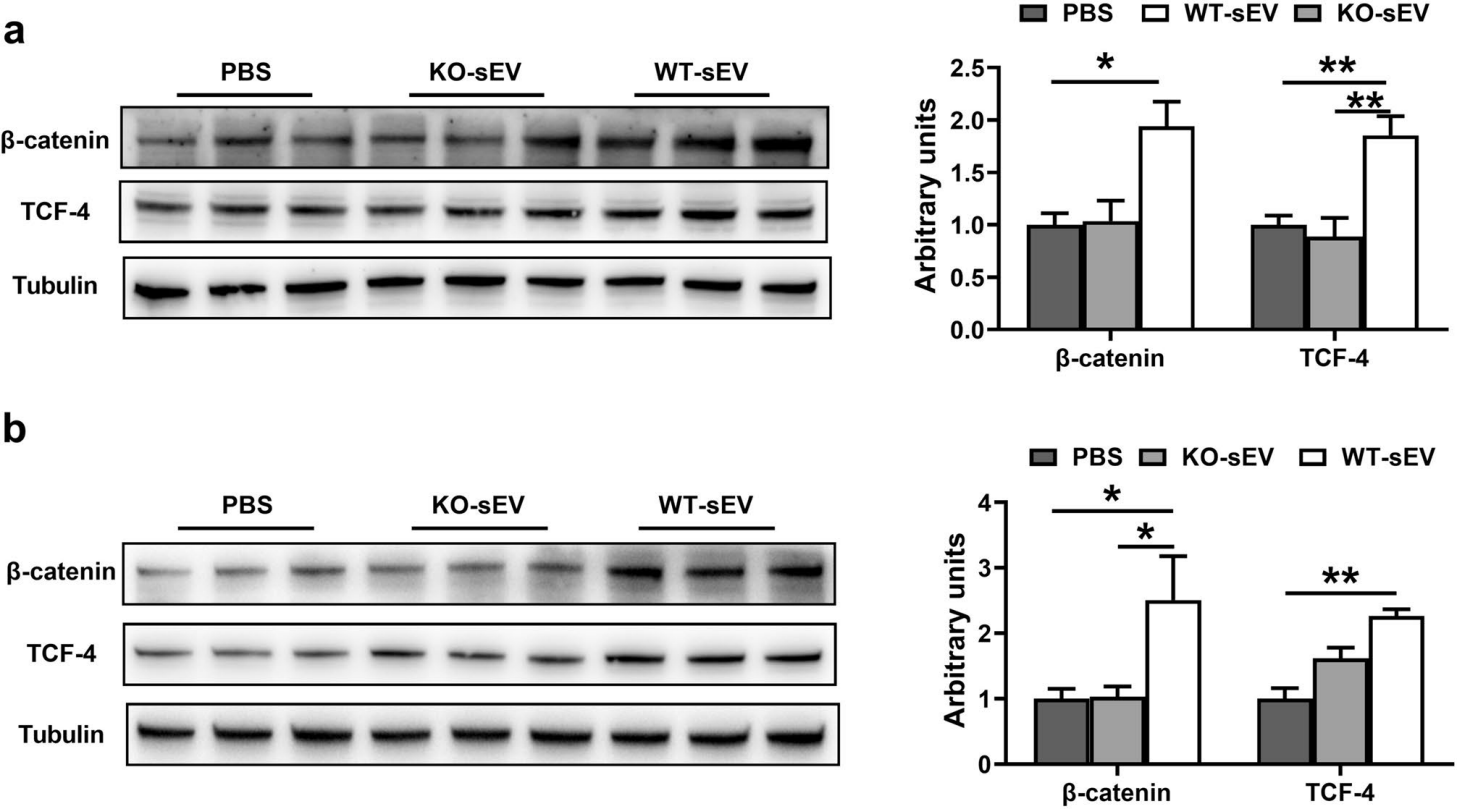
miR-143-3p in pituitary sEV promote liver repair via the Wnt/β-catenin pathway. Western blot analysis of Wnt/β-catenin signaling pathway-related proteins in (**a**) miR-143 KO liver (n = 6), (**b**) Hepatocytes (n = 6).

Previous research has confirmed that IGF-1 is able to stimulate the β-catenin pathway^53^, and tissue repair in the mouse liver following acute CCl_4_ damage depends on injury-induced Wnt/β-catenin signaling^28,47^. Furthermore, it is well known that when Wnt signaling is activated, β-catenin complexes with TCF-4 in the nucleus to induce the TCF-4 transcriptional response^54^. To determine whether miR-143 in pituitary sEV promotes liver repair through this pathway, we further validated the expression of TCF-4. As expected, TCF-4 expression was obviously up-regulated after WT-sEV but not miR-143 KO-sEV treatment (Fig. 7a). We further confirmed this point in liver parenchymal (Fig. 7b). Above results demonstrate that miR-143-3p in pituitary sEV promotes hepatocyte proliferation via the Wnt/β-catenin signaling pathway.

## Discussion

The sEV has been recognized to be significant for remote intercellular communication due to its ability to transfer important cellular cargoes such as mRNA, miRNA, and proteins through circulation^1–6^. Accumulating evidences suggest that EVs are possible key players in endocrine regulation^13–18^. As well known, the pituitary is the central gland of the endocrine system that regulates multiple physiological processes including the cell generation cycle, stress response, growth, reproduction, bone metabolism, and lactation by secreting hormones. Despite the fact that sEV has gained widespread attention as a new important mediator of cellular communications, reports of pituitary sEV have been limited to pituitary adenoma^19–22^ and very rare information is available on sEV from normal pituitary so far.

**Figure 8:**
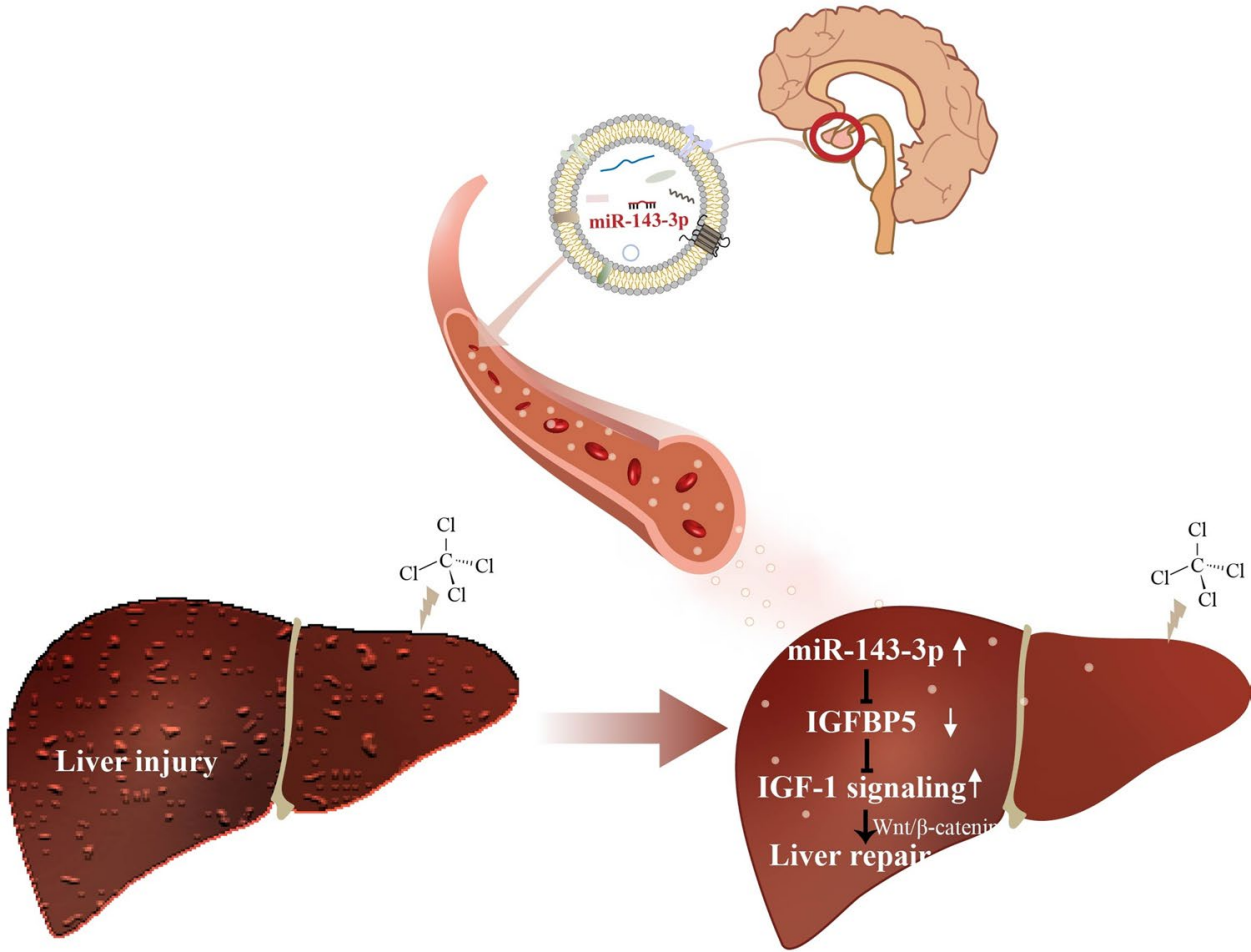
Schematic model of pituitary sEV miR-143-3p regulating liver regeneration and repair.

As well known, the hypothalamic-pituitary–somatotropic axis (HPS axis) plays an essential role in growth regulation. The liver is the key downstream organ of pituitary regulation and plays an important role in metabolism homeostasis. The pituitary gland secretes growth hormone (GH) into the circulation and stimulates IGF-1 production in the liver^55^. However, it has not been illustrated that the pituitary gland regulates the liver via sEV previously. Furthermore, although the liver is notable for its impressive regenerative capacity, liver disease remains a global health problem, especially acute liver injury associated with high mortality rates^56–58^. In response to acute injury or resection, the hepatocyte in a resting state can be triggered to regenerate and restore tissue homeostasis^56,59–62^. Studies have shown that EVs represent a powerful tool to repair tissue damage^63,64^. Furthermore, sEV has been demonstrated to be a potential tool in liver diseases as a diagnostic, prognostic and therapeutic biomarker^65–67^. In addition, numerous studies have showed that sEV derived from liver and mesenchymal stem cells can serve as a promising new class of therapeutic biological particles capable of promoting liver regeneration and repair^24–29^. Furthermore, a previous study has suggested that the pituitary adenoma cell GH1-derived sEV attenuated malignant HCT116 cell motility in vitro^21^. In the present study, for the first time to our knowledge, we demonstrate that the pituitary gland-derived sEV promotes hepatocyte proliferation and liver repair by its cargo miR-143-3p (Fig. 8).

In this study, pituitary sEV derived from MMQ cells and mouse pituitary gland were collected after ultracentrifugation as guided by MISEV2018^31–33^ and confirmed by TEM, NTA and Western blotting. Subsequently, CCK8, EdU, and the immune blot of proliferation marker PCNA revealed that MMQ-sEV was able to promote BRL 3A cell proliferation. More interestingly, sEV from the mouse pituitary gland also exhibited promotion to primary mouse liver parenchymal cell. Which components in pituitary sEV conduct this promotion is the key to understanding its mechanism. Numerous studies showed that sEV played a modulatory role by transferring miRNA to the target cells and sEV-derived miRNAs have generated wide attention considering the minute quantities of miRNAs have dramatic effects^8–12,68^. Intriguingly, miR-143-3p in MMQ-sEV was the most highly expressed miRNA revealed by PCR detection. Besides, the level of miR-143-3p in swine anterior pituitary sEV was also relatively high in our previous study^23^, indicating its important role in pituitary sEV. An elevated level of miR-143-3p was also detected within cells after MMQ-sEV treatment. Further research showed that miR-143-3p inhibitor was able to reverse the facilitation effect on cell proliferation of MMQ-sEV in vitro, suggesting the importance of miR-143-3p in promoting proliferation by sEV. In addition, other miRNAs in sEV may have similar effects and alternative mechanism may exist in response to the treatment with MMQ-sEV. Based on the RNA-seq, we found that many other miRNAs like miR-148a-3p, miR-21-5p and miR-122-5p could regulate the expression of PCNA and CyclinD1^69–74^.

To further verify this point, CRISPR/CAS9 technology was used to generate a miR-143 KO mouse model. Then, we investigated the in vivo effect of pituitary sEV by intravenously administering sEV after inducing acute liver injury via CCl_4_ in miR-143 KO murine model. CCl_4_ is widely used to induce acute liver injury in animals, which serves as a well-established murine model that resembles acute chemical liver injury in humans, to investigate potential therapeutic strategies^75–80^. Strikingly, only WT-sEV treatment, but not miR-143 KO-sEV, significantly reduced ALT and AST levels in the serum of liver-injured miR-143 KO mice, indicating amelioration of liver injury, since serum aminotransferase levels (ALT and AST) are two of the critical parameters to assess liver function damage^81^. Simultaneously, WT-sEV treatment significantly enhanced hepatocyte proliferation and relieved liver damage caused by CCl_4_. Our data first confirmed that pituitary sEV possesses the ability to promote hepatocyte proliferation by delivering miR-143-3p to target cells (Supplementary Information).

Moreover, there were 33 identical target genes of miR-143-3p among the three online bioinformatics tools. Finally, we focused on *Igfbp5* which is involved in cell proliferation. IGBP5 is a negative regulator of IGF-1 which can promote hepatocyte proliferation, differentiation, cell cycle progression and prolong survival^45–48^. Furthermore, it has already been shown that miR-143-3p regulates cell proliferation via IGF-1 signaling by targeting IGFBP5^49^. Our study also confirmed that miR-143-3p mimics promoted cell proliferation by upregulating the expression of IGF-1 through downregulating the expression of IGFBP5. Furthermore, in miR-143 KO mouse, the protein expression of PCNA and IGF-1 were significantly lower, while IGFBP5 was higher, than those in WT mice, no matter detected in vitro or in vivo. A study suggests that IGF-1 is able to stimulate the β-catenin pathway^53^ and Wnt signaling regulates proliferation following acute CCl_4_ injury^82^. As expected, our detection showed that the expression of proteins related to the Wnt/β-catenin pathway also cleared upregulation after WT-sEV treatment. Similar results were obtained in hepatocytes, further confirming miR-143 promotes liver cell proliferation by the IGFBP5-IGF1-Wnt/β-catenin pathway.

## Conclusion

In conclusion, the current study opens a new window to describe the role of the pituitary gland in regulating the liver via sEV. Our findings demonstrate that the beneficial effect of pituitary sEV was tied to the delivery of miR-143-3p to hepatocytes, who activated IGF-1 signaling by targeting IGFBP5 followed by activation of the Wnt/β-catenin pathway, resulting in promoting hepatocyte proliferation. Importantly, our results describe a novel and significance of pituitary sEV in a novel regulation way of the pituitary-liver axis and open a new field for endocrine regulation.

## Data Availability

The raw sequence reads have been deposited in the NCBI Sequence Read Archive (SRA) repository with the accessions SRS13011913. BioProject accession number is SAMN28422839.
